# Supplementary material for: Exercise-induced enhancement of synaptic function triggered by the inverse BAR protein, Mtss1L
Source: eLife. 2019 Jun 24;8:e45920. doi: 10.7554/eLife.45920 (PMC6609409; doi:10.7554/eLife.45920)
Supplement: Supplementary file 1. [file elife-45920-supp1.docx]

| **Supplementary File 1**  **Figure 2: Measurement** | **tdT-** | **tdT+** | **Number of** | **Statistical** | **p-value** |
| --- | --- | --- | --- | --- | --- |
| Input Resistance (MΩ) | 541 ± 105 | 449 ± 73.7 | **pairs (mice)**  13 (8) | **Test**  Paired t-test | 0.4425 |
| Cell Capacitance (pF) | 63.4 ± 5.8 | 64.4 ± 5.2 | 13 (8) | Paired t-test | 0.7703 |
| Series Resistance (MΩ) | 21.4 ± 1.6 | 21.5 ± 2.5 | 13 (8) | Paired t-test | 0.9644 |
| **Figure 6:** |  |  |  |  |  |
| **Measurement**  Input Resistance (MΩ) | **tdT-**  933 ± 77.0 | **tdT+/GFP+**  568 ± 113 | **Number of pairs (mice)**  7 (5) | **Statistical Test**  Paired t-test | **p-value**  0.0792 |
| Cell Capacitance (pF) | 63.2 ± 6.3 | 73.5 ± 8.7 | 7 (5) | Paired t-test | 0.3140 |
| Series Resistance (MΩ) | 17.7 ± 0.9 | 14.9 ± 2.6 | 7 (5) | Paired t-test | 0.3966 |
| **Figure 6—figure supplement 1:** |  |  |  |  |  |
| **Measurement**  Input Resistance (MΩ) | **tdT-**  815 ± 108 | **tdT+/GFP+**  1041 ± 226 | **Number of pairs (mice)**  6 (3) | **Statistical Test**  Paired t-test | **p-value**  0.3915 |
| Cell Capacitance (pF) | 55.8 ± 6.5 | 60.5 ± 5.4 | 6 (3) | Paired t-test | 0.6196 |
| Series Resistance (MΩ) | 18.7 ± 1.8 | 17.8 ± 2.9 | 6 (3) | Wilcoxon | 0.5625 |

**Supplementary File 1**. Intrinsic properties of granule cells during paired recordings.
