# Supplementary material for: Exercise-induced enhancement of synaptic function triggered by the inverse BAR protein, Mtss1L
Source: eLife. 2019 Jun 24;8:e45920. doi: 10.7554/eLife.45920 (PMC6609409; doi:10.7554/eLife.45920)
Supplement: Supplementary file 4. [file elife-45920-supp4.docx]

| **Supplementary File 4**  **Sequence Name**  Ubc-F  Ubc-R  18S-F  18S-R | **Unit**  0.025  0.025  0.025  0.025 | **Size**  21  21  20  20 | **Bases Sequence**  GACGTACCTTCCTCACCACAG  TAAGACACCTCCCCCATCACA  CGCGGTTCTATTTTGTTGGT  TCGTCTTCGAAACTCCGACT |
| --- | --- | --- | --- |
| Elavl4 set1-F | 0.025 | 22 | GAT TCA GGC TGG ACA ATT TGC T |
| Elavl4 set1-R | 0.025 | 21 | CCA GGG ATG TTC ATT CCC ACA |
| Elavl4 3'-F | 0.025 | 20 | TTC CCA TGC TGA ACC CAC TA |
| Elavl4 3'-R | 0.025 | 20 | GCA GCC TCG GTT TTC GTT AT |
| Mtss1l-F | 0.025 | 20 | CCT TCC GAC ATC ACC AGC CA |
| Mtss1l-R | 0.025 | 20 | CTG AGG GTT GTT CGT GGG GA |
| Tnfrsf12a-F | 0.025 | 20 | ATC CTC GTG TTG GGA TTC GG |
| Tnfrsf12a-R | 0.025 | 20 | TCC ATG CAC TTG TCG AGG TC |
| Rgs12-F | 0.025 | 20 | TAG GGA TCC CCG CCT TTC AA |
| Rgs12-R | 0.025 | 20 | CAT CGG CTC TGT TGC TCT GA |
| Vgf-F | 0.025 | 22 | ACA CGC TGA CCC GAG TGA ATC T |
| Vgf-R | 0.025 | 22 | CAT ACG CGC CTG GAA TTG AGA G |
| Stmn2-F | 0.025 | 20 | CAA CTT CAG CAA GAT GGC GG |
| Stmn2-R | 0.025 | 20 | TCC TTG TTC CTG CGA ACC TC |
| Egr1-F | 0.025 | 20 | TTC AAT CCT CAA GGG GAG CC |
| Egr1-R | 0.025 | 20 | TAA CTC GTC TCC ACC ATC GC |
| Cdkn1a-F | 0.025 | 20 | CTG AGC GGC CTG AAG ATT CC |
| Cdkn1a-R | 0.025 | 20 | ACC AAT CTG CGC TTG GAG TG |

**Supplementary File 4:** List of primers used for RT-qPCR analysis.
